# Cost-Effectiveness Analysis of Prostate Health Index in Decision Making for Initial Prostate Biopsy

**DOI:** 10.3389/fonc.2020.565382

**Published:** 2020-11-24

**Authors:** Da Huang, Xiaoqun Yang, Yishuo Wu, Xiaoling Lin, Danfeng Xu, Rong Na, Jianfeng Xu

**Affiliations:** ^1^ Department of Urology, Ruijin Hospital, Shanghai Jiao Tong University School of Medicine, Shanghai, China; ^2^ Department of Pathology, Ruijin Hospital, Shanghai Jiao Tong University School of Medicine, Shanghai, China; ^3^ Department of Urology, Huashan Hospital, Fudan University, Shanghai, China; ^4^ Program for Personalized Cancer Care, NorthShore University HealthSystem, Evanston, IL, United States

**Keywords:** prostate health index, prostate biopsy, cost-effectiveness, cutoff, China

## Abstract

**Background:**

Clinical studies have suggested that prostate health index (*phi*) outperforms prostate-specific antigen (PSA) tests in prostate cancer detection. The cost-effectiveness of *phi* with different cutoffs is poorly understood in the context of decision making for prostate biopsy.

**Methods:**

In a multicenter cohort, 3,348 men with elevated total PSA (tPSA) underwent initial prostate biopsy from August 2013 to May 2019. We constructed a decision model to evaluate the incremental cost-effectiveness ratios of different *phi* cutoffs. Total costs and reimbursement payments were based on the fee schedule of Shanghai Basic Medical Insurance and converted into United States dollars ($). Two willingness-to-pay thresholds were estimated as one or three times the average gross domestic product per capita of China ($7,760 or $23,279, respectively).

**Results:**

The total costs of prostate biopsy and PSA tests were estimated at $315 and $19, respectively. The cost of *phi* test varied between $72 to $130 in different medical centers. Under different *phi* cutoffs (from 23 to 35), *phi* test predicted reductions of 420 (21.7%) to 972 (50.2%) in unnecessary biopsies, with a total gain of 23.77–57.58 quality adjusted life-years compared to PSA tests. All the cutoffs would be cost-effective for patients with tPSA levels of 2–10 ng/ml. Applying 27 as the cutoff was cost-effective for each tPSA range, with missing positive cases ranging from 11 (3.4%) to 33 (11.5%).

**Conclusions:**

Using *phi* test was cost-effective in the decision-making process for initial prostate biopsy, especially for patients with tPSA values between 2–10 ng/ml. The *phi* cutoff of 27 was cost-effective regardless of tPSA ranges and should be recommended from a health-economic perspective.

## Introduction

Prostate-specific antigen (PSA) screening has been widely implemented in decision making for prostate biopsy in clinical practice. However, its low specificity causes large numbers of negative, unnecessary biopsies (1, 2), resulting in physiological and psychological burdens on patients (3). It also results in millions of dollars of unnecessary medical expenditures. To solve these clinical predicaments, novel indicators, such as the prostate health index (*phi*), derived from total PSA (tPSA), free PSA (fPSA), and [-2]proPSA (p2PSA), have been introduced. P*hi* has shown significantly better accuracy in predicting prostate cancer (PCa) than tPSA or %fPSA (fPSA/tPSA). It is now recommended by clinical guidelines for patients with elevated tPSA, especially for patients with tPSA between 2.0 and 10.0 ng/ml (4–7). Synthesizing several published results, using *phi* can reduce the number of unnecessary biopsies with good sensitivity and detect clinically significant PCa; thus, *phi* might play an important role reducing overdiagnosis and overtreatment in urologic practice (8, 9).

The cost of *phi* testing varies among different countries and regions from $35 to $370 (United States dollars) (9–14). Several cost-effectiveness analyses of *phi* have been performed in the United States, Europe, and Hong Kong, which are all based on the population-based screening models using a specific *phi* cutoff (11, 14–16). However, *phi* is mainly applied in men scheduled to undergo prostate biopsy (also known as the prostate biopsy cohort). The cost-effectiveness of *phi* application has never been reported in a biopsy cohort. In addition, differences in subsequent treatment options and medical insurance systems among countries make it difficult to apply the cost-effectiveness evaluation from previous studies to healthcare policy in mainland China. Therefore, we conducted the present study to evaluate the comparative cost-effectiveness using different cutoffs of *phi* in a multicenter biopsy cohort in mainland China.

## Materials and Methods

### Study Population

In this prospective observational multicenter study, we consecutively recruited 3,504 patients from August 2013 to May 2019 in seven tertiary hospitals in Shanghai, China. All the patients underwent initial prostate biopsies. The indications for prostate biopsy were the same across different tertiary hospitals: (1) a tPSA level >10.0 ng/ml; (2) a tPSA level >4.0 ng/ml with confirmation after 2–3 months; (3) %fPSA <0.16 when patients had a tPSA level >4.0 ng/ml; and (4) the presence of suspicious lesions detected by digital rectal examination (DRE), ultrasound or magnetic resonance imaging (MRI) at any level of tPSA. The *phi* calculation was not used in clinical decision making. MRI was not used for biopsy decision making in patients with a tPSA level >4.0 ng/ml.

Blood samples were collected prior to biopsies on the same day and were measured in a central certified lab. Transrectal ultrasound (TRUS)-guided biopsies were performed using a 10- to 14-core scheme. All biopsy specimens were independently examined by 2 experienced pathologists in the department of pathology at each hospital. Clinically significant PCa (csPCa) was defined as PCa with Grade Group (GG) ≥2. The study was approved by the institutional review board of each hospital, and written informed consent was obtained from each participant.

Patients were excluded from the present study if (1) information in the pathological report was missing, (2) serum antigen levels (tPSA, fPSA, or p2PSA) were unable to be tested due to poor serum sample quality, or (3) the tPSA level was <2.0 ng/ml.

### Cost

Currently, application of *phi* (including tPSA, fPSA and p2PSA) has not been approved by the local Health Commission (but has been approved by the China Food and Drug Administration). Thus, the test fee was not fixed among medical centers. The price of *phi* test differed based on mutual agreements between each medical center and the certified lab. Therefore, in the present study, the cost of applying *phi* was assumed based on the information from the certified lab, with a range from $72 to $130 (**Table 1**). Additional costs associated with the prostate biopsy procedure were estimated based on the fee schedule of Shanghai Basic Medical Insurance (SBMI) for employees and residents. We calculated the average reimbursement among the patients and entered it into our cost-effectiveness model (**Table 1**). As described above, *phi* test had not been included in the reimbursement lists of SBMI. We assumed that *phi* test would have the same reimbursement percentage as prostate biopsy in our analysis.

**Table 1:** Estimated total costs and reimbursement used in cost-effectiveness model.

Charge list	Costs (CNY)	Costs (USD)
PSA tests, range^*^	128	19
*phi* test, range	500–900	72–130
Biopsy procedure		
Ultrasonography	385	56
Prostate Biopsy	779	113
Medicine^**^	111	16
Pathological tests	900	130
Total	2,175	315
Reimbursement, mean (SD)	1,781 (305)	258 (44)

PSA, prostate-specific antigen; phi, prostate health index; CNY, Chinese yuan; USD, United States dollar.

^*^PSA tests included both tPSA and fPSA test.

^**^Medicine included both anesthetic and antibiotic drugs.

All the costs noted above were reported in Chinese currency (CNY) and converted to United States dollar using the average exchange rate ($1 = 6.90 CNY) in 2019 (17). We evaluated the cost-effectiveness when making decisions at a single time point; therefore, the impacts of inflation and the annual discount rate were not considered in the analyses.

### Utilities

We multiplied the utility estimates (ranging between 0 and 1, representing death and perfect health, respectively) and duration of health states to calculate the loss of QoL. We assigned a utility estimate of prostate biopsy of 0.90, and its duration of 3 weeks according to published studies (18, 19). Both life-years and quality-adjusted life-years (QALYs) were gained due to the reduction in unnecessary biopsies.

### Outcomes

The primary outcome (necessary biopsy) was pathologically confirmed PCa. In contrast, unnecessary biopsies were defined as negative biopsy results. When considering different *phi* cutoffs, the number of unnecessary biopsies avoided was defined as a “true negative” number (the *phi* value was below the threshold and no cancer was indicated on biopsy). Similarly, a missing positive case was defined as a “false negative” result (the *phi* value was below the threshold but PCa was confirmed). The percentage of unnecessary biopsies avoided and missing positive cases was calculated as the number of unnecessary biopsies avoided and missing positive cases divided by the number of total negative biopsies and PCa, respectively. The secondary outcome was csPCa, and in this case, both indolent PCa (GG = 1) and negative results were classified as unnecessary biopsies.

### Statistical Analysis

The derivative variable *phi* was calculated as follows: (p2PSA/fPSA) ×√tPSA. Since all patients with tPSA levels <2.0 ng/ml were excluded, the remaining patients were recommended to receive prostate biopsies mainly because of elevated tPSA levels. By assuming that men would receive or not receive prostate biopsies under a certain *phi* cutoff in the cohort (and make hypothetical biopsy decisions thereafter), we were able to compute the incremental costs and QALYs.

The incremental costs were calculated as follows: the cost of the [*phi* test] × N—the costs of [prostate biopsy] × n (N: the number of total biopsy times, n: the number of unnecessary biopsies under a certain *phi* cutoff). The incremental effectiveness (QALYs gained) was calculated as follows: the number of biopsies avoided × utility estimate × duration.

Eventually we calculated the incremental cost-effectiveness ratios (ICERs) of different *phi* cutoffs by dividing the incremental costs by the incremental QALYs. With each *phi* cutoff, both the incremental cost and ICER had a range depending on the unit price of *phi* test and the reimbursement percentage. Two willingness-to-pay (WTP) thresholds were estimated as one or 3 times the average gross domestic product (GDP) per capita of China from 2013 to 2018 (20) ($7,760 and $23,279 per QALY, respectively) on the basis of the World Health Organization (WHO) recommendation (21). Thus, it would be considered very cost-effective if the ICER was less than $7,760/QALY; cost-effective if the ICER was between $7,760 and $23,279/QALY gained; and not cost-effective for the remainder.

We evaluated four *phi* cutoffs from 23 to 35 according to the results from two previous studies (based on a Chinese population and a multi-institutional cohort) (7, 22). The cutoff threshold was defined as the number above which all *phi* cutoffs were cost-effective regardless of the unit price of *phi* test. The price threshold indicated that the unit price of *phi* test should be lower than that number to meet the WTP thresholds with each *phi* cutoff.

Sensitivity analyses were also performed by varying the range of tPSA levels. Continuous variables were tested by Student’s t-test for normally distributed variables, and Mann-Whitney U test for non‐normally distributed variables. All statistical analyses were performed using Stata 15.1 Special Edition (StataCorp, 4905 Lakeway Drive, College Station, Texas 77845 USA, 2017). A two-tailed *P* value < 0.05 was considered statistically significant.

## Results

The characteristics and biopsy outcomes of the entire cohort and subgroups are shown in **Table 2** and **Table S1**. Based on our exclusion criteria, 156 patients were excluded, and 3,348 patients were included for further analysis. In the entire cohort, 1,411 (42.1%) patients had PCa documented on biopsy, and 1,145 (34.2%) had csPCa. As shown in **Table 3**, *phi* tests reduced the number of unnecessary biopsies, with reductions ranging from 420 (21.7%) to 972 (50.2%) under different *phi* cutoffs (from 23 to 35). The number of missing positive cases ranged from 39 (2.8%) to 140 (9.9%). In patients with a tPSA level of 2–10 ng/ml, the number of unnecessary biopsies avoided ranged from 276 (28.5%) to 613 (63.4%), with the missing positive cases ranging from 19 (6.6%) and 74 (25.7%).

**Table 2:** Characteristics and biopsy outcomes of entire cohort and subsets grouped by total prostate-specific antigen.

Variables	Entire cohort	tPSA 2–10ng/ml				tPSA 10.1–20 ng/ml				tPSA 20.1–50 ng/ml		
		PCa	Non-PCa	*P*		PCa	Non-PCa	*P*		PCa	Non-PCa	*P*
Patients, n (%)	3,348 (100)	288 (23.0)	967 (77.1)	/		373 (36.9)	639 (63.1)	/		319 (55.8)	253 (44.2)	/
	Median (IQR)					Median (IQR)				Median (IQR)		
Age, year	68 (62–74)	70 (64–75)	65 (60–70)	<0.001		71 (64–76)	66 (61–71)	<0.001		71 (66–77)	68 (63–73)	<0.001
tPSA, ng/ml	12.9 (8.1–25.8)	7.5 (6.0–8.7)	7.0 (5.3–8.5)	<0.001		13.9 (11.9–16.6)	13.3 (11.7–16.0)	0.031		29.6 (24.2–37.5)	25.2 (22.1–30.8)	<0.001
fPSA, ng/ml	1.8 (1.1–3.4)	0.9 (0.7–1.2)	1.1 (0.7–1.5)	<0.001		1.6 (1.1–2.2)	1.9 (1.3–2.7)	<0.001		2.8 (1.9–4.0)	3.4 (2.2–5.0)	<0.001
p2PSA, pg/ml	22.1 (13.0–52.1)	16.4 (11.3–23.6)	12.2 (8.3–18.1)	<0.001		28.4 (18.3–45.7)	19.6 (13.4–27.2)	<0.001		55.5 (32.7–105.7)	30.6 (21.3–48.9)	<0.001
f/tPSA	0.13 (0.09–0.19)	0.13 (0.09–0.18)	0.17 (0.12–0.22)	<0.001		0.11 (0.08–0.15)	0.14 (0.10–0.19)	<0.001		0.09 (0.07–0.13)	0.13 (0.08–0.18)	<0.001
p2/fPSA	14.4 (9.5–23.1)	18.5 (13.3–24.4)	11.7 (8.5–16.3)	<0.001		18.4 (13.2–27.5)	10.3 (7.5–14.9)	<0.001		21.3 (15.1–31.0)	9.8 (6.8–14.5)	<0.001
*phi*	48.5 (29.8–106.4)	49.1 (34.5–68.1)	29.5 (21.7–41.0)	<0.001		69.2 (48.9–102.5)	38.6 (27.7–54.6)	<0.001		114.6 (78.6–174.0)	49.6 (35.0–74.1)	<0.001

PCa, prostate cancer; IQR, interquartile range; tPSA, total prostate-specific antigen; fPSA, free prostate-specific antigen; p2PSA, [-2]proPSA; phi, prostate health index.

**Table 3:** Clinical endpoints, incremental effectiveness and ICER in entire cohort and patients with tPSA values between 2 and 10 ng/ml.

Cutoff values	# Missing positive cases (%)	# Unnecessary biopsies avoided (%)	QALY gained (life-years)	ICER of total cost (USD per QALY)	ICER of reimbursement part (USD per QALY)
Entire cohort (n = 3,348, PCa = 1,411, non-PCa = 1,937)					
*phi* = 23	39 (2.8)	420 (21.7)	23.77	8,203	7,647
*phi* = 27	63 (4.5)	609 (31.4)	34.80	3,674	3,643
*phi* = 31	100 (7.1)	802 (41.4)	46.71	1,185	1,443
*phi* = 35	140 (9.9)	972 (50.2)	57.58	-189	229
tPSA 2–10 ng/ml (n = 1,255, PCa = 288, non-PCa = 967)					
*phi* = 23	19 (6.6)	276 (28.5)	15.28	2,247	2,383
*phi* = 27	33 (11.5)	400 (41.4)	22.42	-409	35
*phi* = 31	53 (18.4)	528 (54.6)	30.08	-1,856	-1,244
*phi* = 35	74 (25.7)	613 (63.4)	35.57	-2,508	-1,821

ICER, incremental cost-effectiveness ratio; tPSA, total prostate-specific antigen; phi, prostate health index; QALY, quality-adjusted life-year; USD, United States dollar; PCa, prostate cancer.

The total costs of prostate biopsy were estimated at $315, with details shown in **Table 1** (slight differences may exist due to the different antibiotic prescriptions, so the number was the average cost from 100 randomly selected patients). The fixed cost of PSA tests (including tPSA and fPSA) was $19. The cost range of *phi* tests for the three components, as described, was $72–$130. Compared to PSA tests, *phi* test led to a total gain in QALYs that ranged from 23.77 to 57.58 life-years due to the reduction in unnecessary biopsies, and 15.28–35.57 life-years in the subgroup with tPSA levels of 2–10 ng/ml (**Table 3**).

**Figure 1:**
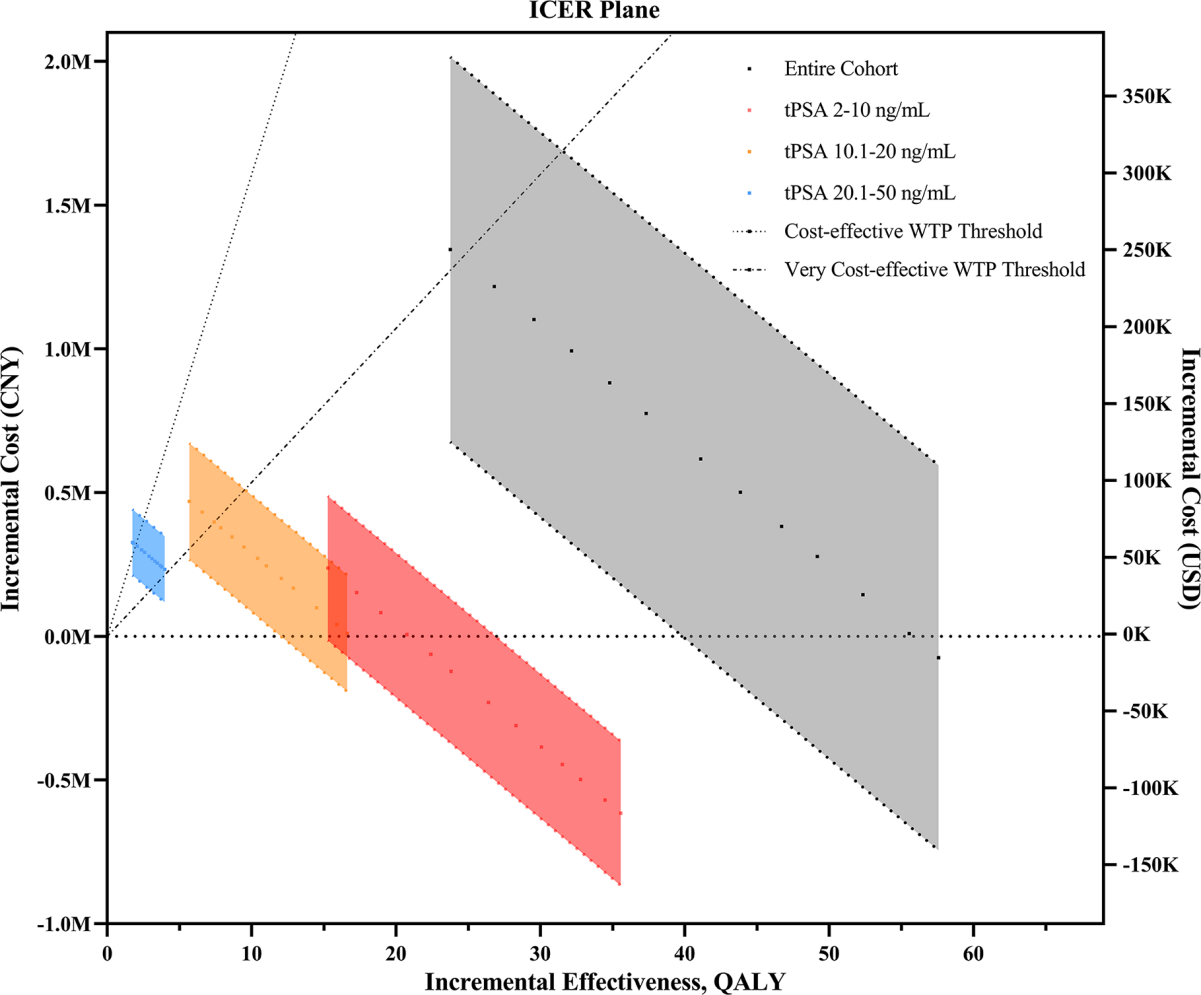
The ICER plane for the comparison of phi- and PSA-based prostate biopsy models upon changes in different *phi* cutoff values. ICER, incremental cost-effectiveness ratio; CNY, Chinese yuan; USD, United States dollar; QALY, quality-adjusted life years; tPSA, total prostate-specific antigen; WTP, willingness-to-pay.

The comparative ICERs between different *phi* cutoffs were evaluated. Higher cutoffs were associated with lower incremental costs and ICERs in all subsets grouped by tPSA values (**Figure 1**). In the entire cohort, the cost-effectiveness analysis showed that all *phi* cutoffs were cost-effective, and *phi* cutoffs over 27 were considered very cost-effective. Notably, when using 27 (or above) as the cutoff, *phi* test would be cost-effective at tPSA range, with limited missing positive cases varying from 11 (3.4%) to 33 (11.5%, **Table 3** and **Table S2**).

In patients with tPSA levels of 2–10 ng/ml, *phi* test would be cost-effective regardless of the cutoffs and unit price. It would be very cost-effective if *phi* cutoffs of 23 or above were applied. Using cutoffs over 27 would even save the total costs (the incremental cost was less than zero). Similar results were found when evaluating the incremental costs of reimbursement parts (**Table 3**). Therefore, under the most accepted *phi* cutoff (as 28) in China, as reported in a previous study (7), *phi* test would be very cost-effective for biopsy decision making. In addition, *phi* test with a cutoff over 27 strictly dominated PSA tests due to negative ICERs (indicating higher effectiveness but less cost). In patients with tPSA levels of 10.1–20 ng/ml, however, *phi* test with a cutoff of 23 or higher were cost-effective regardless of the unit price (**Table S2** and **Table S3**). In patients with tPSA levels of 20.1–50 ng/ml, the lowest cutoffs of 23 was extended dominance (more effective and more costly but higher than our WTP threshold). Similarly, the price thresholds varied from $90 to $122 with cutoffs ranging from 23 to 26 (**Table S3**), and *phi* tests with a cutoff of 27 or higher were cost-effective regardless of the unit price.

Assuming that the cost of *phi* tests would be reimbursed proportionally at the same percentage as prostate biopsy, similar results were found from an insurance perspective (**Table 3** and **Table S2**). All the cutoffs would be very cost-effective in patients with tPSA levels of 2–10 ng/ml. Cutoffs over 27 were cost-effective at each tPSA range regardless of the unit price. Repeated analysis showed similar results after using csPCa as the biopsy outcome (**Table S4**).

The expected incremental costs, also known as out-of-pocket payments, were also compared between different groups, as shown in **Figure S1A** (when the total cost of *phi* test was paid by the patients themselves) and **Figure S1B** (when the cost of *phi* test was reimbursed by SBMI). The expected incremental costs decreased with increased cutoffs in all subsets grouped by tPSA range. Furthermore, the out-of-pocket payment for *phi* test was lower in patients with tPSA values between 2–10 ng/ml than in the other two subgroups (all *P* values < 0.001).

## Discussion

The objective of the present study was to evaluate whether *phi* test was cost-effective in decision making for initial prostate biopsy. In this multicenter study, we found that *phi* test was not only more accurate for PCa detection, but also more cost-effective than PSA tests, especially for patients with tPSA levels of 2–10 ng/ml in the Chinese population. In addition, applying 27 as *phi* cutoff would be very cost-effective in different tPSA subgroups, with very limited missing positive cases. The results from the present study suggested that *phi* test had health-economic advantages and could even save costs for national medical insurance in China.

Four published studies have demonstrated that *phi* test is cost-effective in screening models of developed regions (11, 14–16). However, all the previous studies inputted single cutoffs for *phi* test (25 and 35 for Western and Chinese populations, respectively) into their models and analyzed data in several screening cycles. Furthermore, the WTP thresholds ($50,000/QALY) in previous studies were much higher than those in our study ($7,760 or $23,279/QALY) because of socioeconomic differences. Regarding the costs of *phi* testing, a nonfixed price from $72 to $130 was allocated in our study, which was close to the price used in European and US models (10, 11, 15, 16), but much less than that in a Hong Kong model ($369.54) (14). Therefore, the results of previous studies may not be generalizable to our population. In the present study, we evaluated the ICERs of different *phi* cutoffs and performed subgroup analyses according to tPSA values. We found that applying 27 as *phi* cutoff was cost-effective regardless of tPSA range and unit price, with limited missing positive cases. In mainland China, basic medical insurance is provided nationwide and is nonprofitable. As a result, the high incidence of PCa and high reimbursement percentage of prostate biopsy increase the economic burden in the whole country. Hence, the present results are remarkable for developing countries such as China, where *phi* test is still not included in the national medical insurance reimbursement list.

The present study focused on only the cost-effectiveness at the diagnostic stage (in which men with elevated tPSA levels would undergo prostate biopsy). We did not take the subsequent treatments into account due to the homogeneous treatment patterns for PCa in mainland China. Zhao et al. (23) reported that only 2.33% of low-risk PCa patients received active surveillance (AS) or observation in China, even though AS is widely recommended by the guidelines for low-risk patients (24, 25). In other words, most indolent PCa patients in China actually receive curative treatment, and more than 80% of them undergo radical prostatectomy rather than radiation therapy (23). Several factors might explain the above differences. For example, the cultural background makes it hard to tolerate a malignancy without a dissection; the relatively low accessibility of persistent health care and follow-up service is another critical driven factor for the high rate of curative treatments (26). Therefore, the overtreatment effects caused by *phi* testing in the present study would be very low (natural overtreatment due to the disease management). For the same reason, we considered PCa rather than csPCa as the main outcome in the current study, as it was more suitable for our biopsy cohort in the Chinese population.

There were several limitations of this study. First, we evaluated the medical expenditures associated with *phi* test by using a hypothetical reimbursement percentage. However, the serum test and biopsy are both classified into the same reimbursement list in China, and the reimbursement percentage varies by age and socioeconomic status rather than medical usage. Thus, the hypothetical percentage might be reliable in our analysis. Second, the health utilities used in our model were from published articles of Western populations, due to a lack of related research in China. However, prostate biopsy is a short-term procedure that might vary little between Western and Asian populations. Third, the WTP threshold based on the GDP was used to evaluate the cost-effectiveness in each situation; however, the WTP was uncertain and lacked specificity (27, 28). We added the reimbursement percentage into our decision model to adjust for the budget impact. However, further analyses are needed to assess the long-term impact of *phi* test. Fourth, all medical centers participating in the present study were located in Shanghai, a large city in East China, which may cause selection bias. However, individuals all over the country seek the services of these tertiary hospitals.

In conclusion, *phi* test was cost-effective in decision making for initial prostate biopsy in the Chinese population, especially patients with tPSA values between 2–10 ng/ml. The *phi* cutoff of 27 was cost-effective regardless of the tPSA ranges, with limited missing positive cases, and should be recommended from a health-economic perspective.

## Data Availability Statement

The raw data supporting the conclusions of this article will be made available by the authors, without undue reservation.

## Ethics Statement

The studies involving human participants were reviewed and approved by The institutional review board of Ruijin Hospital, Huashan Hospital, Cancer Center, Xinhua Hospital, Huadong Hospital, Ninth People’s Hospital, and Changhai Hospital in Shanghai. The patients/participants provided their written informed consent to participate in this study.

## Author Contributions

RN and DX conceived and designed the study. DH, XY, YW, XL, and JX contributed materials and collected the data. DH and XY analyzed the data. DH, XY, RN, and DX wrote the manuscript. All authors contributed to the article and approved the submitted version.

## Funding

This work was supported by grants from the National Natural Science Foundation of China (Grant No. 81772741 and No. 81972645), Shanghai Rising-Star Program (Grant No. 18QA1402800), the “Chen Guang” project from Shanghai Municipal Education Commission and Shanghai Education Development Foundation (Grant No. 17CG09), Shanghai Jiao Tong University School of Medicine Gaofeng-Clinical Medicine Grant Support (Grant No. 20181701), Shanghai Municipal Human Resources and Social Security Bureau (Grant No. 2018052), and Shanghai Jiao Tong University SMC-Chenxing Scholar Project to RN. All the funders had no role in the study design, data collection, data analysis, interpretation, and writing of the report.

## Conflict of Interest

In the present study, we declare that Beckman Coulter, Inc. provided the tests for tPSA, fPSA, and p2PSA. All the sample collection, data analyses, and manuscript writing were performed by the researchers, independent from Beckman Coulter, Inc.

The authors declare that the research was conducted in the absence of any commercial or financial relationships that could be construed as a potential conflict of interest.
