# Supplementary material for: Cost-Effectiveness Analysis of Prostate Health Index in Decision Making for Initial Prostate Biopsy
Source: Front Oncol. 2020 Nov 24;10:565382. doi: 10.3389/fonc.2020.565382 (PMC7732507; doi:10.3389/fonc.2020.565382)

Supplementary Material

**Table S1** Characteristics and biopsy outcomes (Grade Group ≥ 2) of entire cohort and subsets grouped by total prostate‑specific antigen

**Table S2** Clinical endpoints, incremental effectiveness and ICER in patients with tPSA values between 10.1-50 ng/mL

**Table S3** The comparative price thresholds (USD) between different phi cutoffs in entire cohort and subsets grouped by total prostate-specific antigen

**Table S4** Clinically significant endpoints, incremental effectiveness and ICER in entire cohort and patients with tPSA values between 2-10 ng/mL

**Figure S1** The expected incremental costs (out-of-pocket payment) of different phi cutoffs in entire cohort and subsets grouped by tPSA: (A) all the cost of phi tests was paid by patients themselves; (B) the cost of phi tests would be reimbursed partly by medical insurance. The error bars represented the upper and lower limit values.

**Table S1** Characteristics and biopsy outcomes (Grade Group ≥ 2) of entire cohort and subsets grouped by total prostate‑specific antigen

| Variables | Entire cohorts | tPSA 2-10ng/mL | | |  | tPSA 10.1-20 ng/mL | | |  | tPSA 20.1-50 ng/mL | | |
| --- | --- | --- | --- | --- | --- | --- | --- | --- | --- | --- | --- | --- |
|  |  | GG ≥ 2 | Others | *P* |  | GG ≥ 2 | Others | *P* |  | GG ≥ 2 | Others | *P* |
| Patients, n (%) | 3,348 (100) | 185 (14.7) | 1,070 (85.3) | / |  | 280 (27.7) | 732 (72.3) | / |  | 267 (46.7) | 305 (53.3) | / |
|  | median (IQR) | | |  |  | median (IQR) | |  |  | median (IQR) | |  |
| Age, year | 68 (62-74) | 71 (65-76) | 65 (60-71) | <0.001 |  | 71 (65-76) | 66 (61-72) | <0.001 |  | 71 (66-77) | 68 (63-73) | <0.001 |
| tPSA, ng/mL | 12.9 (8.1-25.8) | 7.5 (5.8-8.6) | 7.0 (5.4-8.5) | 0.038 |  | 14.2 (12.1-16.7) | 13.3 (11.6-16.0) | 0.001 |  | 30.0 (24.2-38.2) | 25.7 (22.2-32.0) | <0.001 |
| fPSA, ng/mL | 1.8 (1.1-3.4) | 0.9 (0.7-1.3) | 1.1 (0.7-1.5) | 0.003 |  | 1.5 (1.1-2.2) | 1.9 (1.3-2.6) | <0.001 |  | 2.9 (1.9-4.0) | 3.2 (2.2-4.9) | 0.008 |
| p2PSA, pg/mL | 22.1 (13.0-52.1) | 16.4 (11.7-26.1) | 12.5 (8.4-18.6) | <0.001 |  | 29.4 (19.5-47.2) | 20.1 (13.7-29.4) | <0.001 |  | 61.1 (34.4-107.1) | 31.7 (21.7-51.2) | <0.001 |
| f/tPSA | 0.13 (0.09-0.19) | 0.13 (0.09-0.18) | 0.16 (0.12-0.22) | <0.001 |  | 0.11 (0.08-0.15) | 0.14 (0.10-0.19) | <0.001 |  | 0.09 (0.07-0.13) | 0.12 (0.08-0.18) | <0.001 |
| p2/fPSA | 14.4 (9.5-23.1) | 19.9 (14.8-24.9) | 12.1 (8.7-16.7) | <0.001 |  | 19.3 (14.1-28.5) | 10.9 (7.8-15.9) | <0.001 |  | 22.0 (15.7-31.4) | 10.3 (7.1-16.5) | <0.001 |
| *phi* | 48.5 (29.8-106.4) | 51.3 (37.7-69.8) | 30.4 (22.3-42.6) | <0.001 |  | 72.2 (51.1-109.3) | 40.5 (29.0-59.5) | <0.001 |  | 119.2 (88.4-178.1) | 52.8 (37.2-90.1) | <0.001 |

Abbreviations: IQR, interquartile range; tPSA, total prostate-specific antigen; fPSA, free prostate-specific antigen; p2PSA, [-2]proPSA; *phi*, prostate health index; GG, Grade group.

**Table S2** Clinical endpoints, incremental effectiveness and ICER in patients with tPSA values between 10.1-50 ng/mL

| Cutoff values | Missing positive cases (%) | Unnecessary  biopsies avoided (%) | QALY gained (life-years) | ICER of total cost (USD per QALY) | ICER of reimbursement part (USD per QALY) |
| --- | --- | --- | --- | --- | --- |
| tPSA 10.1-20 ng/mL (n = 1,012, PCa = 373, non-PCa = 639) | | | | | |
| *phi* = 23 | 11 (2.9) | 99 (15.5) | 5.70 | 11,937 | 10,948 |
| *phi* = 27 | 16 (4.3) | 151 (23.6) | 8.65 | 5,785 | 5,510 |
| *phi* = 31 | 31 (8.3) | 202 (31.6) | 12.06 | 2,422 | 2,537 |
| *phi* = 35 | 48 (12.9) | 273 (42.7) | 16.62 | 89 | 475 |
| tPSA 20.1-50 ng/mL (n = 572, PCa = 319, non-PCa = 253) | | | | | |
| *phi* = 23 | 6 (1.9) | 28 (11.1) | 1.76 | 26,873 | 24,150 |
| *phi* = 27 | 11 (3.4) | 39 (15.4) | 2.59 | 16,326 | 14,827 |
| *phi* = 31 | 12 (3.8) | 53 (20.9) | 3.37 | 11,153 | 10,255 |
| *phi* = 35 | 14 (4.4) | 63 (24.9) | 3.99 | 8,467 | 7,880 |

Abbreviations: ICER, incremental cost-effectiveness ratio; tPSA, total prostate-specific antigen; phi, prostate health index; QALY, quality-adjusted life-year; USD, United States dollar; PCa, prostate cancer.

**Table S3** The comparative price thresholds (USD) between different phi cutoffs in entire cohort and subsets grouped by total prostate-specific antigen

| Cutoff values | Cost-effective | | | |  | Very cost-effective | | | |
| --- | --- | --- | --- | --- | --- | --- | --- | --- | --- |
|  | Entire Cohort | Subgroup by tPSA (ng/mL) | | |  | Entire Cohort | Subgroup by tPSA (ng/mL) | | |
|  |  | 2-10 | 10.1-20 | 20.1-50 |  |  | 2-10 | 10.1-20 | 20.1-50 |
| *phi* = 23 | * | * | * | 90 |  | 98 | * | 78 | / |
| *phi* = 24 | * | * | * | 93 |  | 111 | * | 90 | / |
| *phi* = 25 | * | * | * | 106 |  | 122 | * | 101 | / |
| *phi* = 26 | * | * | * | 122 |  | * | * | 108 | / |
| *phi* = 27 | * | * | * | * |  | * | * | 118 | / |
| *phi* = 28 | * | * | * | * |  | * | * | 130 | / |
| *phi* = 29 | * | * | * | * |  | * | * | * | 75 |
| *phi* = 30 | * | * | * | * |  | * | * | * | 80 |
| *phi* = 31 | * | * | * | * |  | * | * | * | 81 |
| *phi* = 32 | * | * | * | * |  | * | * | * | 84 |
| *phi* = 33 | * | * | * | * |  | * | * | * | 89 |
| *phi* = 34 | * | * | * | * |  | * | * | * | 92 |
| *phi* = 35 | * | * | * | * |  | * | * | * | 97 |

Abbreviations: tPSA, total prostate-specific antigen; phi, prostate health index.

The cost range of phi test was estimated at 72-130 USD in the present study. The price thresholds indicated that the unit price of phi tests should be lower than the number to meet the WTP thresholds. The asterisks mean that all the prices in the cost range were cost-effective, in the contrast, the slash represented that all were not cost-effective.

**Table S4** Clinically significant endpoints, incremental effectiveness and ICER in entire cohort and patients with tPSA values between 2-10 ng/mL

| Cutoff values | Missing necessary biopsies (%) | Unnecessary  biopsies avoided (%) | QALY gained (life-years) | ICER of total cost (USD per QALY) | ICER of reimbursement part (USD per QALY) |
| --- | --- | --- | --- | --- | --- |
| Entire cohort (n = 3,348, csPCa = 1,145, unnecessary biopsies = 2,203) | | | | | |
| *phi =* 23 | 25 (2.2) | 434 (19.7) | 23.77 | 2,181 | 2,023 |
| *phi =* 27 | 37 (3.2) | 635 (28.8) | 34.80 | -440 | -198 |
| *phi =* 31 | 59 (5.2) | 843 (38.3) | 46.71 | -1,880 | -1,419 |
| *phi =* 35 | 80 (7.0) | 1,032 (46.8) | 57.58 | -2,675 | -2,092 |
| tPSA 2-10 ng/mL (n = 1,255, csPCa = 185, unnecessary biopsies = 1,070) | | | | | |
| *phi =* 23 | 12 (6.5) | 283 (26.4) | 15.28 | -1,265 | -898 |
| *phi =* 27 | 18 (9.7) | 415 (38.8) | 22.42 | -2,802 | -2,200 |
| *phi =* 31 | 27 (14.6) | 554 (51.8) | 30.08 | -3,639 | -2,910 |
| *phi =* 35 | 38 (20.5) | 649 (60.7) | 35.57 | -4,017 | -3,230 |

Abbreviations: ICER, incremental cost-effectiveness ratio; tPSA, total prostate-specific antigen; phi, prostate health index; QALY, quality-adjusted life-year; USD, United States dollar; csPCa, clinically significant prostate cancer.

**Figure S1** The expected incremental costs (out-of-pocket payment) of different phi cutoffs in entire cohort and subsets grouped by tPSA: (A) all the cost of phi tests was paid by patients themselves; (B) the cost of phi tests would be reimbursed partly by medical insurance. The error bars represented the upper and lower limit values.


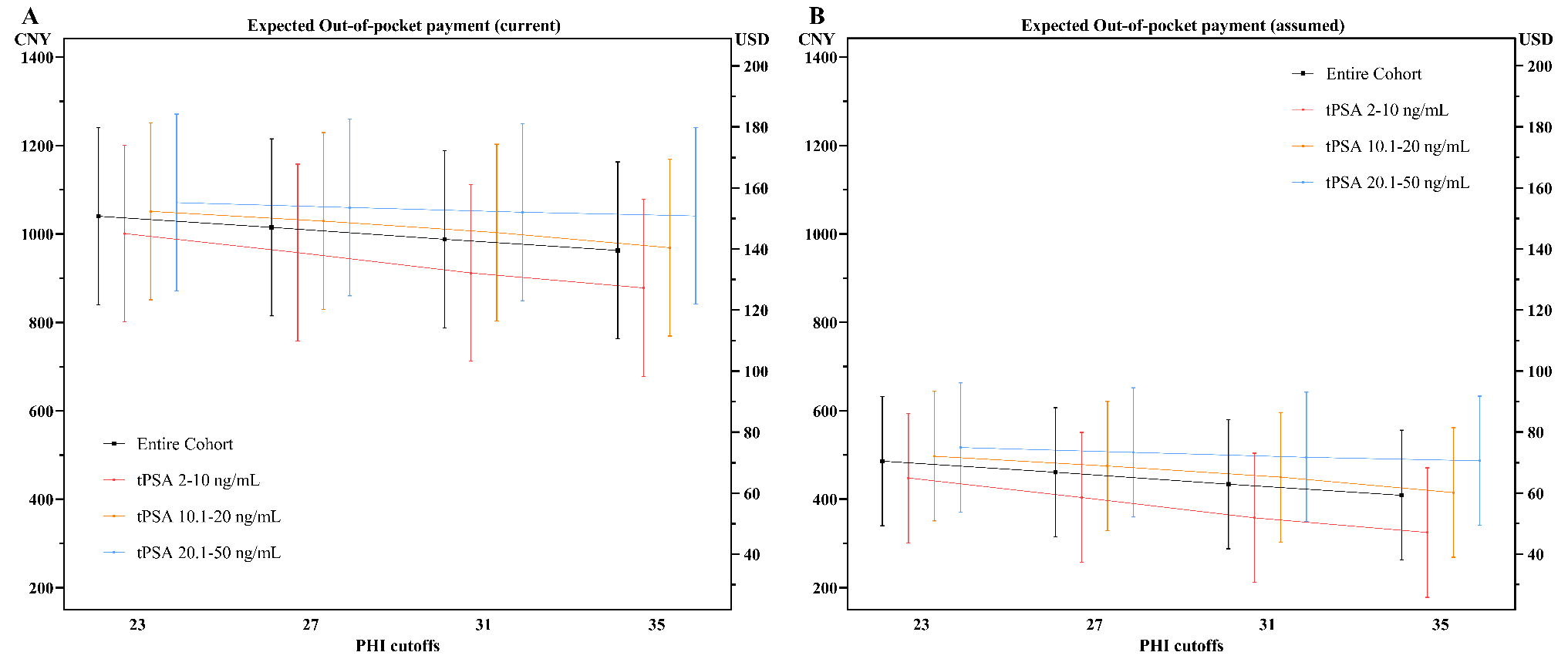

Supplement: Supplementary file 1 [file DataSheet_1.docx]
